# Supplementary material for: Unveiling prognostics biomarkers of tyrosine metabolism reprogramming in liver cancer by cross-platform gene expression analyses
Source: PLoS One. 2020 Jun 15;15(6):e0229276. doi: 10.1371/journal.pone.0229276 (PMC7295234; doi:10.1371/journal.pone.0229276)
Supplement: S1 Fig — Plots were taken from GEPIA online databases (http://gepia.cancer-pku.cn). Data indicates expression after normalization by log2(TMP+1) for comparison between tumor and normal tissues in pan-cancer. The cancer abbreviation names are shown according to TCGA study abbreviations. (DOCX) [file pone.0229276.s001.docx]

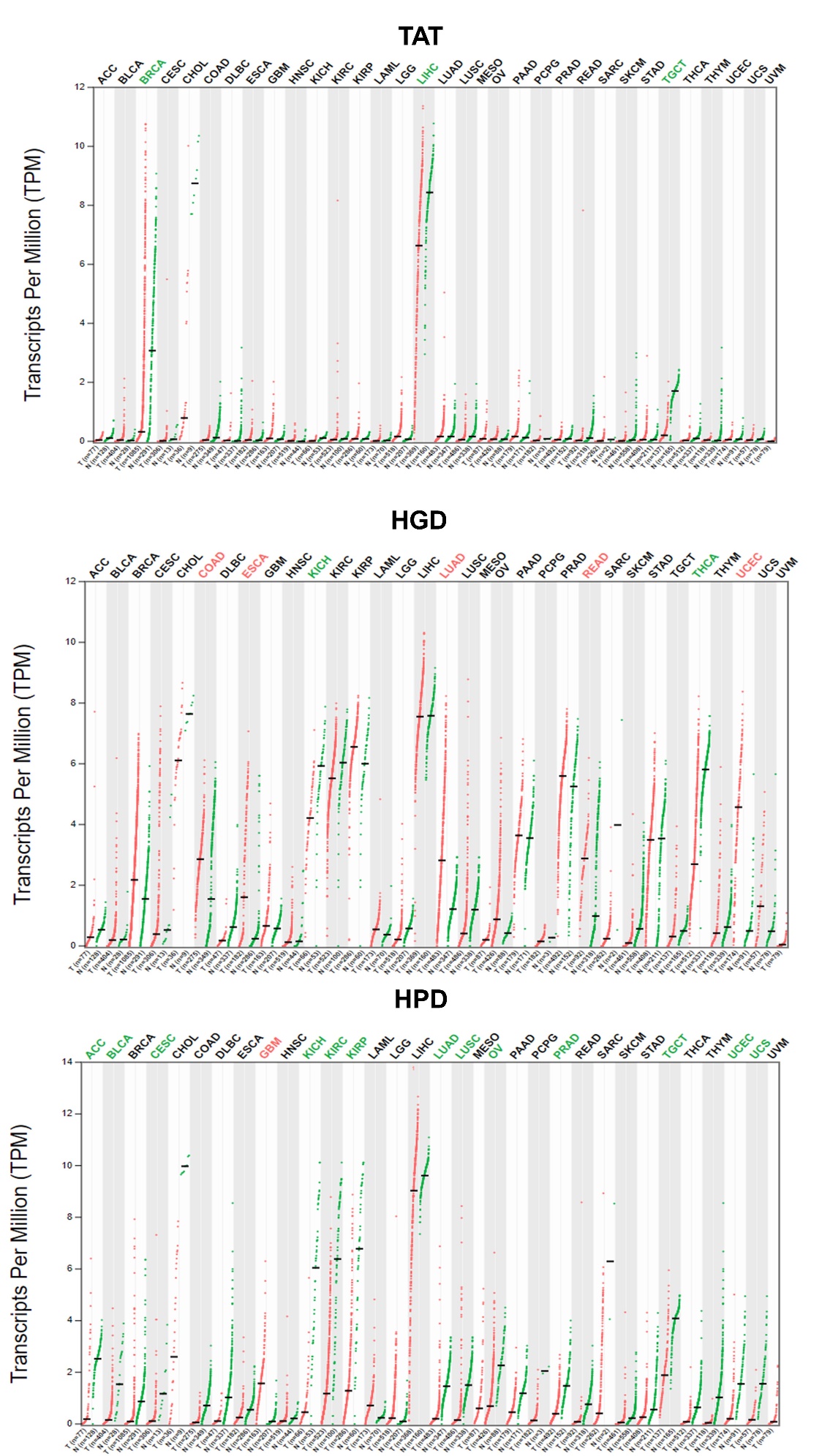


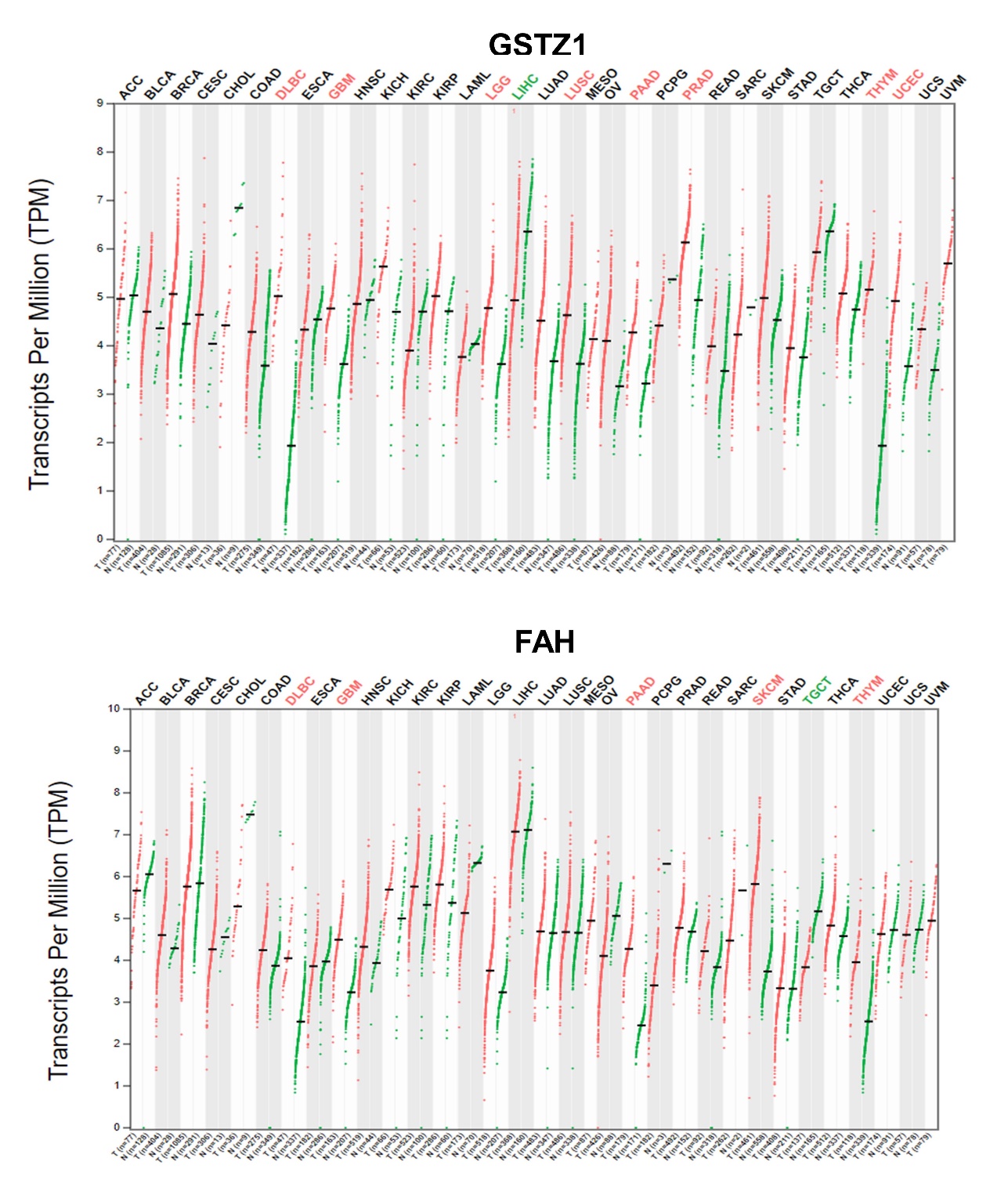


**Figure S1**: **Tyrosine catabolism enzyme-encoding gene expression across TCGA pan-cancer datasets.**

Plots were taken from GEPIA online databases (<http://gepia.cancer-pku.cn>). Data indicates expression after normalization by log_2_(TMP+1) for comparison between tumor and normal tissues in pan-cancer. The cancer abbreviation names are shown according to TCGA study abbreviations.
