# Supplementary material for: Unveiling prognostics biomarkers of tyrosine metabolism reprogramming in liver cancer by cross-platform gene expression analyses
Source: PLoS One. 2020 Jun 15;15(6):e0229276. doi: 10.1371/journal.pone.0229276 (PMC7295234; doi:10.1371/journal.pone.0229276)
Supplement: S2 Fig — Disease-free survival outcomes of 364 HCC patients were analyzed using log-rank tests based on gene expression in HCC tissues from the TCGA cohort. Kaplan-Meier curves are plotted using GEPIA for TAT, HPD, HGD, GSTZ1 and FAH, and HRs and 95% confidence intervals are shown. Abbreviation: HCC, hepatocellular carcinoma, HRs, hazard ratios; TCGA, the Cancer Genome Atlas. (DOCX) [file pone.0229276.s002.docx]

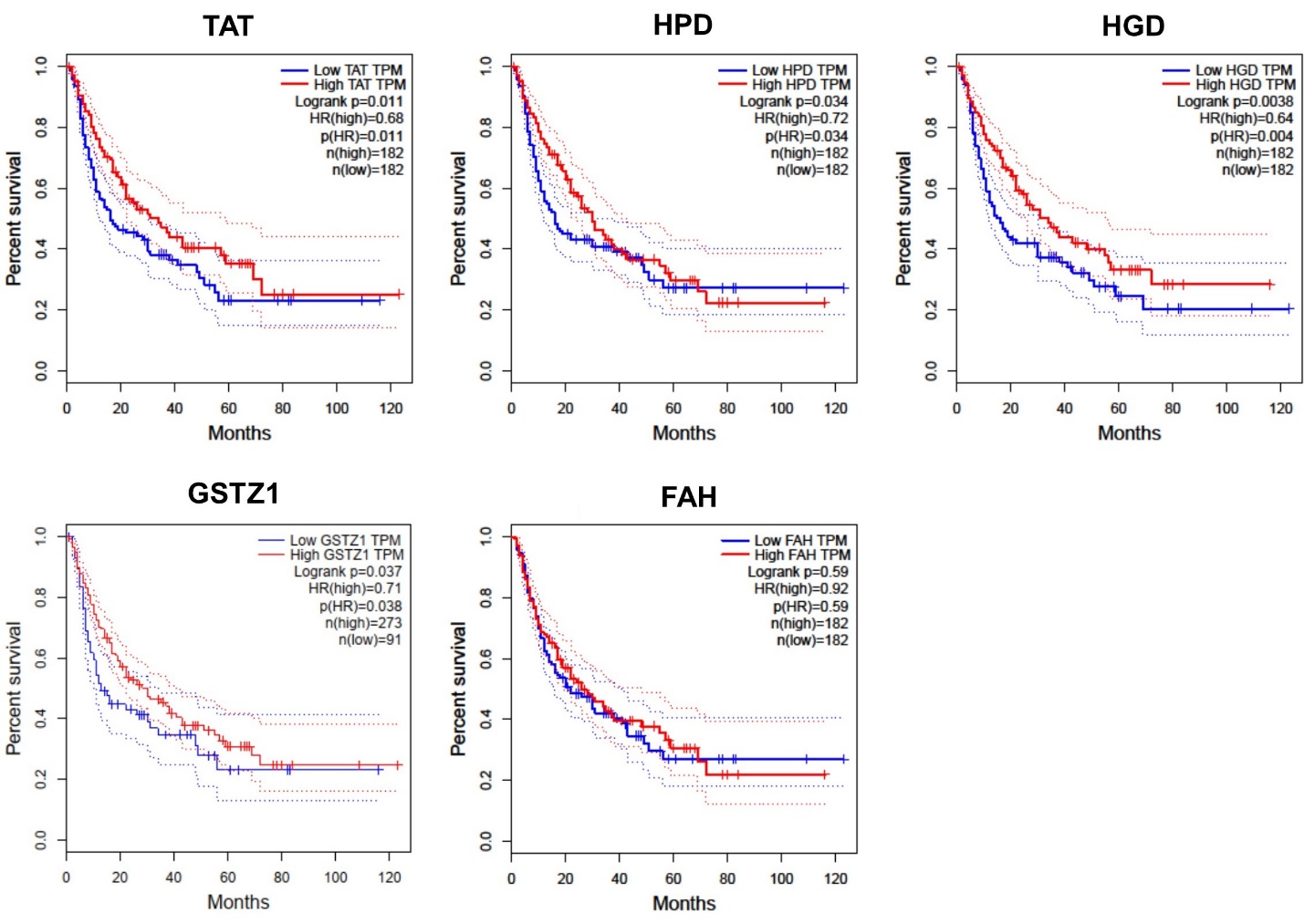


**Figure S2. Disease-free survival outcomes of HCC patients.**

Disease-free survival outcomes of 364 HCC patients were analyzed using log-rank tests based on gene expression in HCC tissues from the TCGA cohort. Kaplan-Meier curves are plotted using GEPIA for TAT, HPD, HGD, GSTZ1 and FAH, and HRs and 95% confidence intervals are shown. Abbreviation: HCC, hepatocellular carcinoma, HRs, hazard ratios; TCGA, the Cancer Genome Atlas.
