# Supplementary material for: Unveiling prognostics biomarkers of tyrosine metabolism reprogramming in liver cancer by cross-platform gene expression analyses
Source: PLoS One. 2020 Jun 15;15(6):e0229276. doi: 10.1371/journal.pone.0229276 (PMC7295234; doi:10.1371/journal.pone.0229276)
Supplement: S3 Fig — GSEA of canonical pathways for differentially expressed genes in GSTZ1 overexpressed liver cancer cells compared to empty vector control. The top twenty significantly enriched canonical pathways (both upregulated and downregulated) were displayed with their corresponding normalized enrichment score. Multiple pathways appear to be related to metabolism, oxidations and cancer development. (DOCX) [file pone.0229276.s003.docx]

*
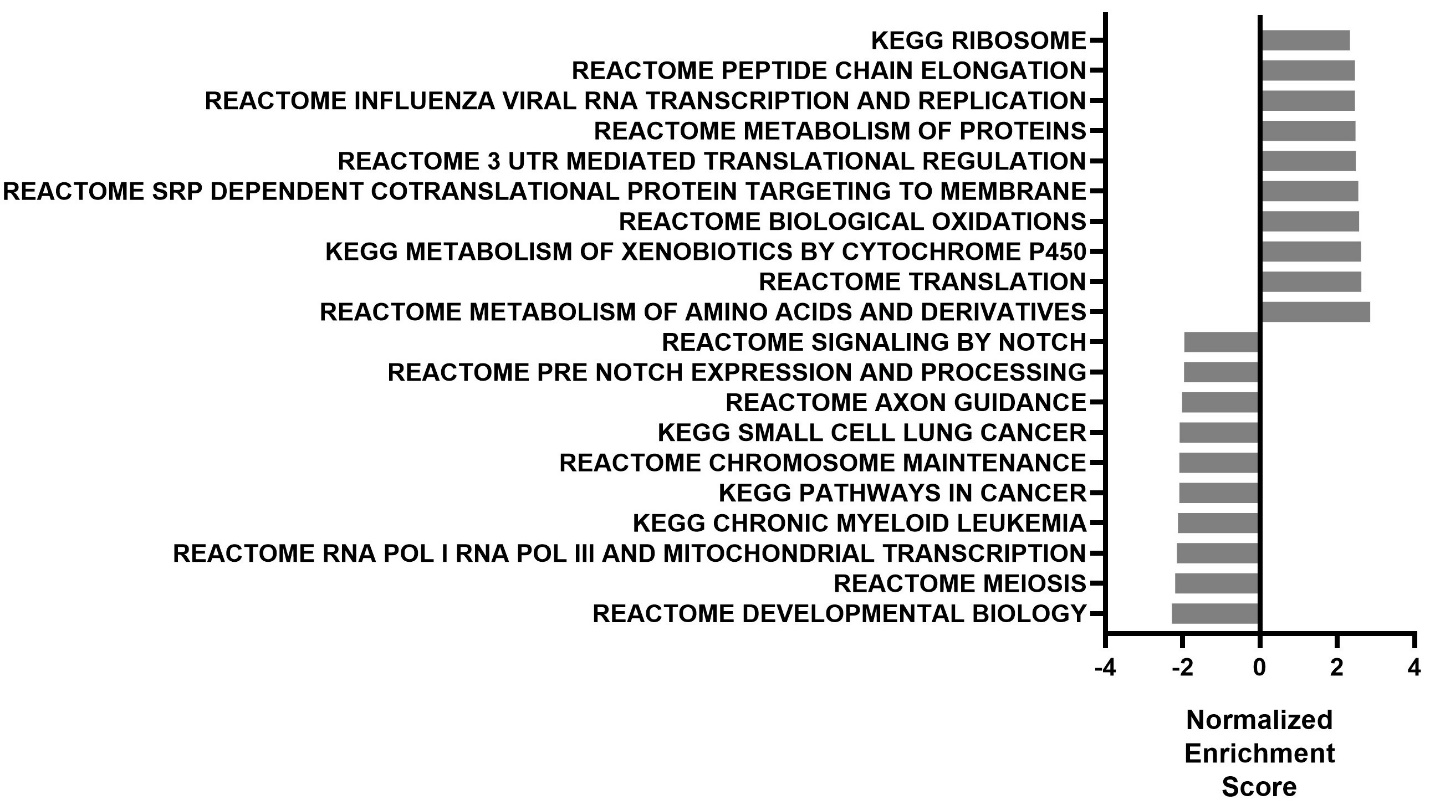
*

**Figure S3**: **Enriched GSEA canonical pathways of differentially expressed genes in GSTZ1 overexpressed liver cancer cells.**

GSEA of canonical pathways for differentially expressed genes in GSTZ1 overexpressed liver cancer cells compared to empty vector control. The top twenty significantly enriched canonical pathways (both upregulated and downregulated) were displayed with their corresponding normalized enrichment score. Multiple pathways appear to be related to metabolism, oxidations and cancer development.
