# Supplementary material for: Unveiling prognostics biomarkers of tyrosine metabolism reprogramming in liver cancer by cross-platform gene expression analyses
Source: PLoS One. 2020 Jun 15;15(6):e0229276. doi: 10.1371/journal.pone.0229276 (PMC7295234; doi:10.1371/journal.pone.0229276)
Supplement: S4 Fig — The mutation profiles of TAT, HPD, HGD, GSTZ1 and FAH was obtained from cBioPortal (Liver hepatocellular Carcinoma, TCGA, PanCancer Atlas, 353 samples). (DOCX) [file pone.0229276.s004.docx]

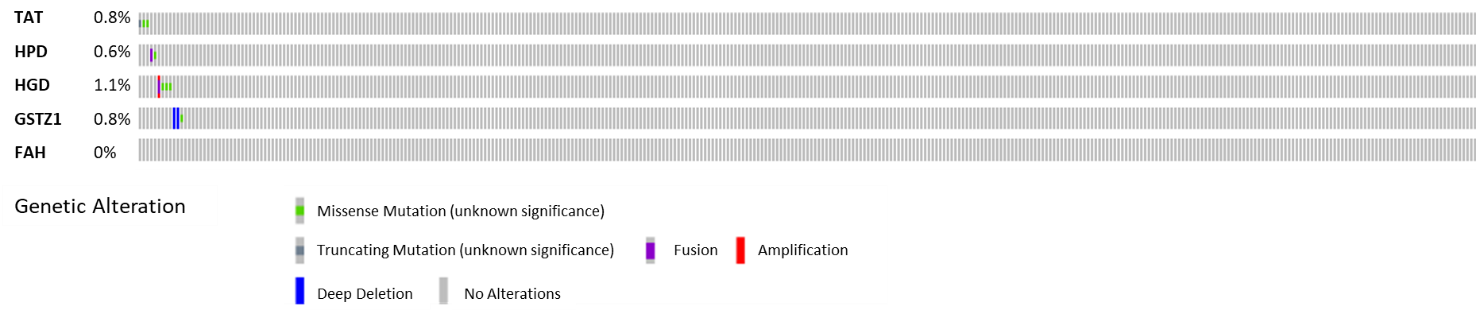


**Figure S4. The mutation profiles of tyrosine catabolic genes.**

The mutation profiles of TAT, HPD, HGD, GSTZ1 and FAH was obtained from cBioPortal (Liver hepatocellular Carcinoma, TCGA, PanCancer Atlas, 353 samples)
