# Supplementary material for: Unveiling prognostics biomarkers of tyrosine metabolism reprogramming in liver cancer by cross-platform gene expression analyses
Source: PLoS One. 2020 Jun 15;15(6):e0229276. doi: 10.1371/journal.pone.0229276 (PMC7295234; doi:10.1371/journal.pone.0229276)
Supplement: S5 Fig — Comparisons of TAT, HPD, HGD, GSTZ1 and FAH relative mRNA expression levels to putative mutation types and relative copy number. Data and plots were obtained from cBioPortal (Liver hepatocellular Carcinoma, TCGA, PanCancer Atlas, 353 samples). (DOCX) [file pone.0229276.s005.docx]

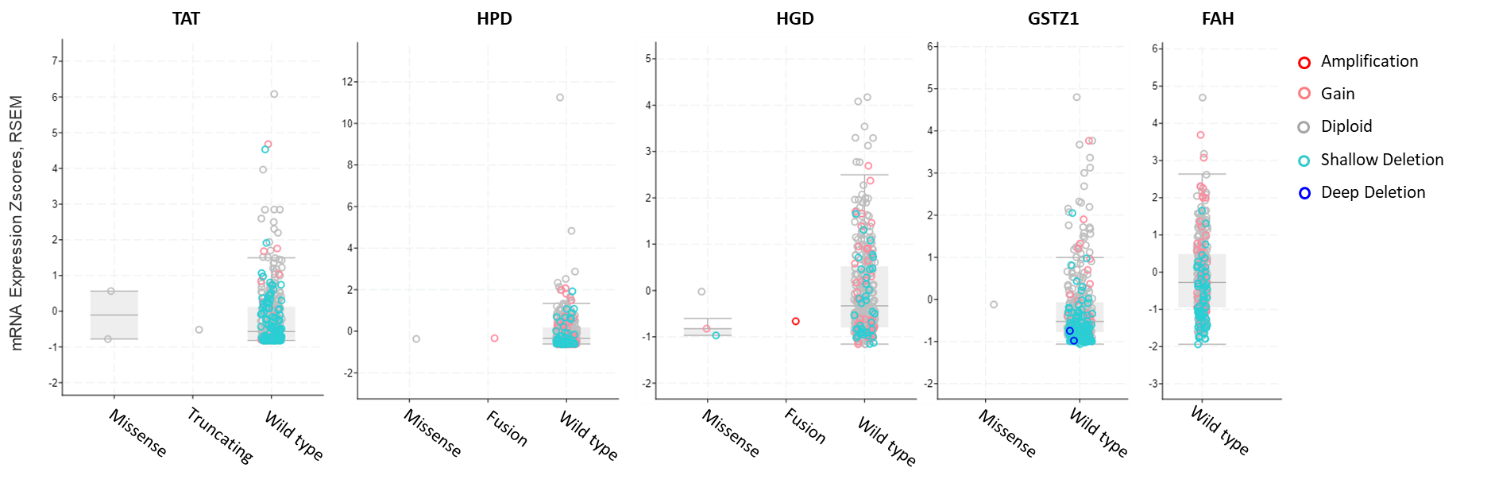


**Figure S5. Gene copy number relative to gene expression of tyrosine catabolic genes.** Comparisons of TAT, HPD, HGD, GSTZ1 and FAH relative mRNA expression levels to putative mutation types and relative copy number. Data and plots were obtained from cBioPortal (Liver hepatocellular Carcinoma, TCGA, PanCancer Atlas, 353 samples).
