# Supplementary material for: Unveiling prognostics biomarkers of tyrosine metabolism reprogramming in liver cancer by cross-platform gene expression analyses
Source: PLoS One. 2020 Jun 15;15(6):e0229276. doi: 10.1371/journal.pone.0229276 (PMC7295234; doi:10.1371/journal.pone.0229276)
Supplement: S6 Fig — The anti-correlation (Pearson correlation: r<0, p<0.05) between miR-539 and TAT, HPD, HGD and GSTZ1 in hepatocellular carcinoma was obtained from starBase35 co-expression analysis on TCGA-LIHC dataset. (DOCX) [file pone.0229276.s006.docx]

*
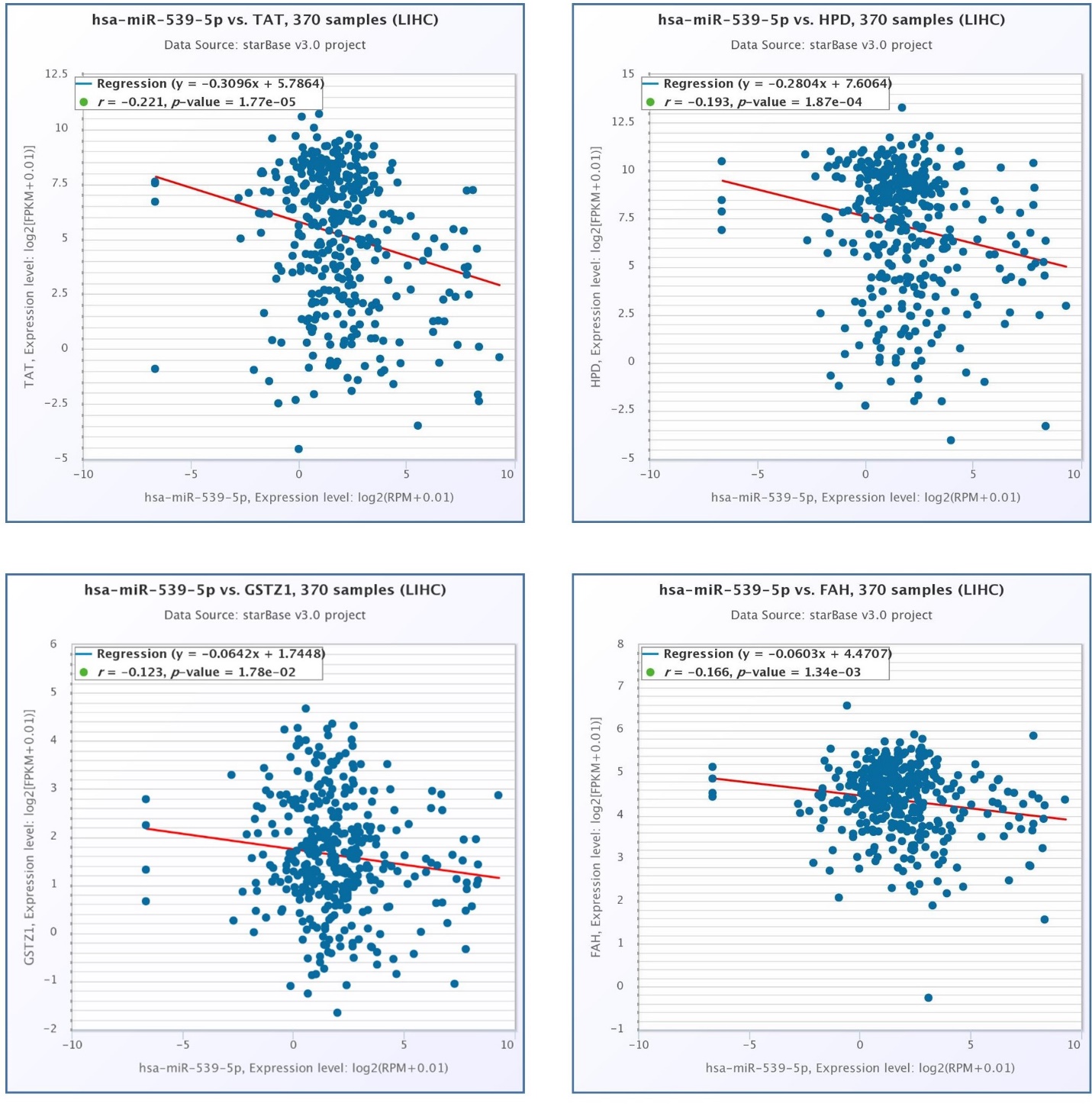
*

**Figure S6. The anti-correlation between miR-539 and tyrosine catabolic genes.**

The anti-correlation (Pearson correlation: r<0, p<0.05) between miR-539 and TAT, HPD, HGD and GSTZ1 in hepatocellular carcinoma was obtained from starBase^35^ co-expression analysis on TCGA-LIHC dataset.
